# Combination Treatment of Hypothermia and Mesenchymal Stromal Cells Amplifies Neuroprotection in Primary Rat Neurons Exposed to Hypoxic-Ischemic-Like Injury *In Vitro*: Role of the Opioid System

**DOI:** 10.1371/journal.pone.0047583

**Published:** 2012-10-15

**Authors:** Yuji Kaneko, Naoki Tajiri, Tsung-Ping Su, Yun Wang, Cesar V. Borlongan

**Affiliations:** 1 Department of Neurosurgery and Brain Repair, University of South Florida College of Medicine, Tampa, Florida, United States of America; 2 National Institute on Drug Abuse, National Institutes of Health, Baltimore, Maryland, United States of America; Stanford University School of Medicine, United States of America

## Abstract

This study was designed to reveal the therapeutic regimen and mechanism of action underlying hypothermia treatment in combination with stem cell transplantation for ameliorating neonatal hypoxic-ischemic-like injury. Primary rat neurons were exposed to oxygen-glucose deprivation (OGD), which produced hypoxic-ischemic-like injury *in vitro*, then incubated at 25°C (severe hypothermia), 34°C (moderate hypothermia), and 37°C (normothermia) with or without subsequent co-culture with mesenchymal stromal cells (MSCs). Combination treatment of moderate hypothermia and MSCs significantly improved cell survival and mitochondrial activity after OGD exposure. The exposure of delta opioid human embryonic kidney cells (HEK293) to moderate hypothermia attenuated OGD-mediated cell alterations, which were much more pronounced in HEK293 cells overexpressing the delta opioid receptor. Further, the addition of delta opioid peptide to 34°C hypothermia and stem cell treatment in primary rat neurons showed synergistic neuroprotective effects against OGD which were significantly more robust than the dual combination of moderate hypothermia and MSCs, and were significantly reduced, but not completely abolished, by the opioid receptor antagonist naltrexone altogether implicating a ligand-receptor mechanism of neuroprotection. Further investigations into non-opioid therapeutic signaling pathways revealed growth factor mediation and anti-apoptotic function accompanying the observed therapeutic benefits. These results support combination therapy of hypothermia and stem cells for hypoxic-ischemic-like injury *in vitro*, which may have a direct impact on current clinical trials using stand-alone hypothermia or stem cells for treating neonatal encephalopathy.

## Introduction

Hypoxic-ischemic encephalopathy (HIE), a subset of neonatal encephalopathy, is a major cause of mortality and severe long-term neurological disorders in infants. The underlying mechanisms of HIE remain poorly understood, but may include hypoxia-ischemia-induced injury [1], aberrant stages of region-specific brain maturation [2], blood brain barrier (BBB) impairment [3], and mitochondrial dysfunction-induced apoptosis [4]. Hypothermia has been shown to afford strong neuroprotective effects against HIE acting on multiple therapeutic pathways with downstream beneficial outcomes such as decreased reactive oxygen species, attenuated BBB breakdown, reduced metabolic ratio, and modified gene/protein expression of inflammation and apoptosis [5]. Mounting evidence has indicated that hypothermia treatment is effective in the first 6 hours of life for the infant with moderate to severe HIE [6]. Although increased survival of newborns diagnosed with neonatal encephalopathy is achieved within this 6-hour window of hypothermia treatment initiation [7], approximately 40% of these babies present with serious neurologic disability [8], [9]. To this end, the current hypothermia treatment protocol may benefit from combination therapeutic strategies [10], [11].

Accumulating experimental data have indicated the mobilization of bone marrow-derived stem cells, such as mesenchymal stromal cells (MSCs), in brain plasticity and therapy of HIE [12]. MSCs are capable of differentiation into variety of tissue specific cells [13], [14], and have been demonstrated to exert therapeutic benefits against brain injury [6]. However, little is known regarding MSC treatment for HIE, especially in combination with hypothermia.

Delta opioids have been implicated in ischemia [15]–[18], and the use of delta opioid agonists may resemble certain physiological correlates of hibernation including hypothermia [15]–[19], which may involve direct opioid receptor activation, as well as non-opioid mechanisms [15], [16], [20]–[22]. To this end, delta opioids may regulate neural stem and progenitor cell proliferation and differentiation [23]–[26], and may even enhance cell-based therapeutics in *in vitro* and *in vivo* disease models [27], [28]. Accordingly, pharmacological investigations targeting delta opioids may reveal unique signaling pathways associated with the neuroprotection conferred by hypothermia and stem cell therapy in the hypoxic-ischemic-like injury disease setting.

We report here that moderate hypothermia is efficacious in an *in vitro* model of hypoxic-ischemic-like injury, which was enhanced by MSC treatment. Equally a novel finding here is that we demonstrate for the first time that the delta opioid system, along with other non-opioid neuroprotective processes, primarily contributes to the observed neuroprotection.

## Materials and Methods

### Experimental Model of Hypoxic-Ischemic-Like Injury *In Vitro*

**Figure 1:**
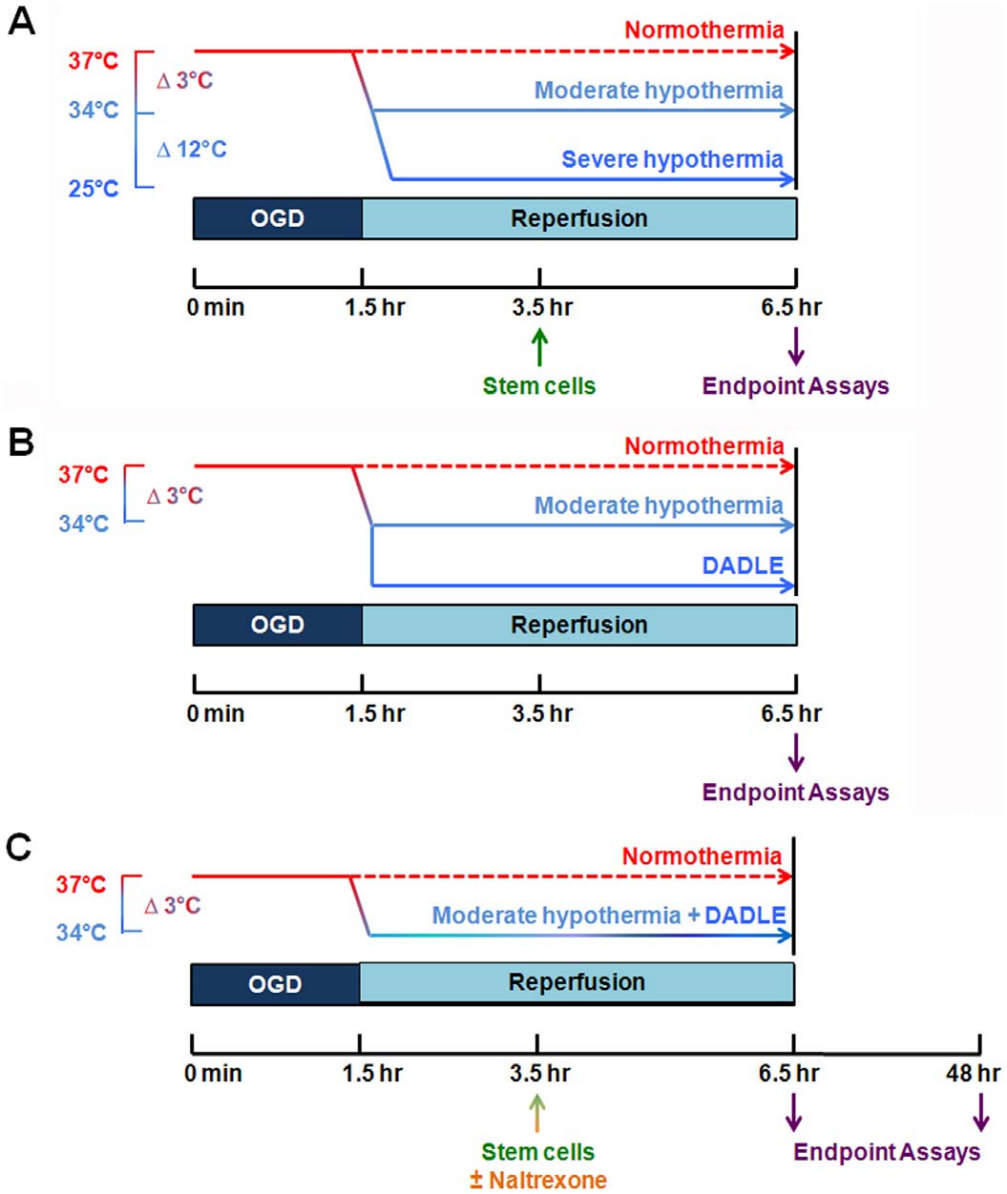
Experimental design. The diagram captures time course of temperature, introduction of stem cells, evaluation of cell viability and mitochondrial activity under different treatment conditions.

Primary rat neuronal cells, PRNCs, (embryos at Day18) were obtained from BrainBits. According to the protocol, cells (4×10^4^ cells/well) were suspended in NbActive4 (BrainBits) in the absence of antibiotics and grown in Poly-L-Lysine-coated 96-well assay plate (BD) at 37°C in humidified atmosphere containing 5% CO_2_ containing 85% neuron and 15% astrocyte cell population (determined immunocytocehmically using vesicular glutamate transpoter-1). After 3 days in culture (a model of a newborn brain), PRNCs were exposed to oxygen glucose deprivation (OGD) as described previously with a few modifications [29]. The cells were initially exposed to OGD medium (116 mM NaCl, 5.4 mM KCL, 0.8 mM MgSO_4_, 1 mM NaH_2_PO_4_, 26.2 mM NaHCO_3_, 0.01 mM glycine, 1.8 mM CaCl_2_ pH 7.4), then placed in an anaerobic chamber (Plas Labs) containing 95% N_2_ and 5% CO_2_ for 15 min at 37°C. Finally, the chamber was sealed and incubated for 90 min at 37°C (hypoxic-ischemic-like injury *in vitro* condition). Control cells were incubated in the same buffer containing 5 mM glucose at 37°C in a regular 5% CO_2_ incubator. OGD was terminated by adding 5 mM glucose to medium and cell cultures re-introduced to the regular CO_2_ incubator atmosphere at 37°C and 95% O_2_ for 5 hours, of which period represented a model of “reperfusion”. The temperatures of reperfusion at 37°C, 34°C, and 25°C were modeled as normothermia, moderate hypothermia, and severe hypothermia, respectively (Figure 1, Panel A). After 2 hours of reperfusion, 2×10^4^ human MSCs were co-cultured with the PRNCs. We caution that the present *in vitro* OGD model may not directly mimic the *in vivo* hypoxic-ischemic condition and warrants further translational research for clinical applications.

### MSC Preparation

Collection, isolation, preparation, and laboratory research use of human MSCs were approved by the Institutional Review Board of Medical College of Georgia. Written informed consent was provided by all donor patients. Briefly, human CD133+ cells were isolated from G-CSF-mobilized leukapheresed blood using magnetic cell sorting technology [30]. Isolated MSCs were centrifuged and resuspended in medium consisting of DMEM with 10% fetal calf serum (FCS) which selected for the growth of MSCs (StemCell Technologies) in the absence of antibiotics. From the initial heterogeneous cell population, CD133+ cells constituted 1%–2%, with the remainder being CD133− cells. Using the harvested CD133+ cell sample, flow cytometry revealed 75%–85% purity for CD133+CD34+ antigens. Approximately, 87% of the selected CD133+ cells were viable upon completion of the selection process [30]. These CD133+ MSCs were kept in culture for 1–2 days prior to the experiment initiation. To avoid the possible bias that MSCs may be more resistant to oxidative stress [31] and to hypoxia [32] than primary neurons, since the stem cell niche is known to be hypoxic [33] thus able to tolerate such non-conducive environment, we ensured that the outcome assays reflected the status of primary neurons instead of MSCs. We note that human MSCs were mostly non-existent during the assay because our short timing of treatment condition did not allow MSCs to attach to the plastic, thus washing of the wells prior to conducting the assays removed the MSCs resulting in our endpoints mostly reflecting the primary rat cell readouts. Finally, we conducted immunocytochemistry of the cell culture system using human specific antigens, which revealed no detectable human MSCs. We, however, highlight that 85% of the cell culture system was neuronal with 15% astrocytic, therefore interpretation of the data should consider these multiple cell types. In particular, the present cell cycle assay was directed at the astrocyte subpopulation which likely displayed phenotypic features of proliferative cells. Finally, based on our initial data demonstrating that the combination therapy was better than stand alone treatment, we reasoned that this combination therapy has more clinically relevant therapeutic benefits over stand-alone treatments thus designed our subsequent experiments in examining the mechanism of action of neuroprotection using this combination therapy.

### Measurement of Cell Viability

Measurement of cell viability was performed by both fluorescent live/dead cell assay [34] and trypan blue exclusion method. A two-color fluorescence cell viability assay was performed by calcein-AM (Invitrogen) retained within live cells, and ethidium homodimer (EthD-1, Invitrogen) bound to the nuclei of damaged cells. Following reperfusion, the cells were incubated with 2 µM calcein-AM and 4 µM EthD-1 for 45 min at room temperature in darkness. Afterwards, cells were washed once with phosphate buffered saline, then the green fluorescence of the live cells was measured by the Gemini EX florescence plate reader (Molecular Device), excitation at 490 nm and emission at 520 nm. In addition, trypan blue (0.2%) exclusion method was conducted and mean viable cell counts were calculated in four randomly selected areas (1 mm^2^, n = 10) to further reveal the cell viability. To precisely calibrate the cell viability, the values were standardized from fluorescence intensity and trypan blue data.

### Measurement of Mitochondrial Activity

Changes in metabolic activity was examined using 3- (4, 5-dimethyl-2-thiazoyl)-2, 5-diphenyltetrazolium bromide (MTT) as previously described [35] and according to manufacturer instructions (Roche, Catalog Number 11465007001). Before completing 2 hours of reperfusion, the cells were incubated with 0.5 mg/ml MTT at 37°C and 5% CO_2_, and incubated with lysis buffer overnight in a humidified atmosphere at 37°C and 5% CO_2_. The optical density of solubilized purple formazan was measured at 570 nm on a Synergy HT plate reader (Bio-Tex).

### Interaction of the Delta Opioid System with Moderate Hypothermia

After validating that moderate hypothermia exerted neuroprotection against hypoxic-ischemic-like injury *in vitro*, we next embarked on pharmacologically determining the mechanism of action underlying this therapeutic outcome. Based on ours and other reports [15]–[19] implicating delta opioids in achieving hypothermic effects, we exposed human embryonic kidney cells (HEK293; ATCC) to moderate (34°C) hypothermia alone, the delta opioid peptide [d-Ala(2), d-Leu(5)]-enkephalin (DADLE, 0.1 nM) alone, or a combination thereof under hypoxic-ischemic-like injury *in vitro* condition. To further reveal the participation of the delta opioid system, HEK293 cells overexpressing the delta opioid receptor (DOR) were generated as previously described and subsequently exposed to the above treatment conditions. HEK293 cells were grown in DMEM under standard conditions. Cells were stably transfected by the calcium phosphate co-precipitation method with plasmid pcDNA 3.1 (Invitrogen) containing full-length cDNA of the DOR. Stably opioid receptor-expressing cell clones were selected with Geneticin (Invitrogen). The cell clones used in this study were designated HEK293DOR which expressed about 1.4 pmol receptors/mg membrane protein comparable to reported expression levels of similar cell clones [36]. HEK293 and HEK293DOR (4×10^4^ cells/well) were subjected to the same timeline of cell culture, treatment initiation and endpoint assays (cell viability and MTT) as detailed above (Figure 1, Panel B). The rationale for using initially the HEK cell line was because of this cell line’s well-characterized DOR-over-expression allowing us to reveal the direct effects of DADLE (i.e., via ligand-receptor modulation) on hypothermia and MSC treatment. Once we confirmed this initial readout of opioidergic mechanism, subsequent experiments were performed using primary neuronal cells.

### Convergence of Moderate Hypothermia, Stem Cell Therapy and Delta Opioid System

Following the lead that moderate hypothermia likely acts on delta opioid system, we further tested this converging therapeutic pathway and examined if additive neuroprotective effects against hypoxic-ischemic-like injury *in vitro* were produced by combination treatment of 34°C hypothermia, MSCs and DADLE (0.1 nM). Here, rather than HEK293 and HEK293DOR cells subsequent experiments used PRNCs exposed to the same timeline of cell culture, treatment initiation and endpoint assays (cell viability and MTT) as detailed above (Figure 1, Panel C).

### Opioid and Non-opioid Mechanism Underlying Hypothermia and Stem Cell Therapy

To reveal whether the therapeutic outcome of combined hypothermia, stem cell therapy and DADLE truly reflected a canonical ligand-receptor mechanism, we used the opioid antagonist naltrexone at a dose (0.1 nM) known to block DADLE effects. In parallel, we investigated non-opioid neuroprotective pathways shown to be associated with DADLE [16], [23]. In addition, to directly determine whether the neuroprotection of hypothermia+MSCs treatment involved an opioid mechanism, we examined the effects of naltrexone on hypothermia+MSCs without DADLE treatment. Supernatants (at the 6.5 hours of treatment, see Figure 1, Panel C) from each treatment condition were collected for enzyme-linked immunosorbent assay (ELISA) using our previously described methods for detecting glial-derived neurotrophic factor (GDNF), nerve growth factor (NGF) and brain-derived neurotrophic factor (BDNF) [16]. After determining that GDNF was significantly upregulated by the combination therapy, we further assessed the role of this growth factor by pre-treating PRNCs with antibody against GDNF [37], [38] in additional cultures exposed to the same treatment conditions as above [37], [38]. Apoptosis was also examined in sister cultures of PRNCS which were plated on Lab-Tek II Chamber Slides (Nalge Nunc International) at a density of 5×10^4^ cells/well. Cells were maintained and treated as described above. Twenty-four hours after each treatment condition, PRNCs were fixed and processed for ApoAlert™ DNA Fragmentation Assay Kit (BD Biosciences. Palo Alto, CA). In the final washing step, cells were incubated with the nuclear dye Hoechst 33342 (1∶1000 from stock at 10 mg/ml; Molecular Probe) for 10 min. Immunofluorescence was detected and processed using a Zeiss Axiophot fluorescence microscope. Finally, the effects of this combination therapy on cell cycle progression were determined by using the FACS analysis of propidium iodide (PI)-stained PRNCs. PRNCs were plated in T75 tissue culture flasks at a density of 10^6^ per flask. Cells in cultures were then synchronized regarding their growth phase by starving them in a serum-free medium for 24 h. At the end of the 24 h, PRNCs were exposed to the treatment conditions. Cells were then maintained in the 0.5% FBS medium for an additional 24 h. Cells were harvested by trypsinization, washed twice with ice-cold PBS, and spun down for 5 min at 300 *g*. The pellets were resuspended in Vindeloves PI staining solution (100 ml Vindeloves PI containing 121 mg Trizma Base, 58 mg NaCl, 5 mg PI, 100 µl NP-40, and 70 Kunitz U RNAse) and stored at 4°C for at least 2 h. PRNCs were then analyzed by flow cytometry by using a FACSCalibur flow cytometer (Becton Dickinson, San Jose, CA). The proportion of cells in the G0/G1, S, and G2/M fractions of the cell cycle was determined by using the ModFit LT software (Verity Software, Topsham, ME).

### Statistical Analysis

ANOVA was used to reveal significant treatment effects followed by Fisher’s PLSD posthoc pairwise comparisons with statistical significance set at p<0.05. Data are represented as mean ± SE from quintuplicates of each treatment condition.

## Results

### Combination Therapy of Moderate Hypothermia and MSCs Preserves Cell Viability Following OGD

**Figure 2:**
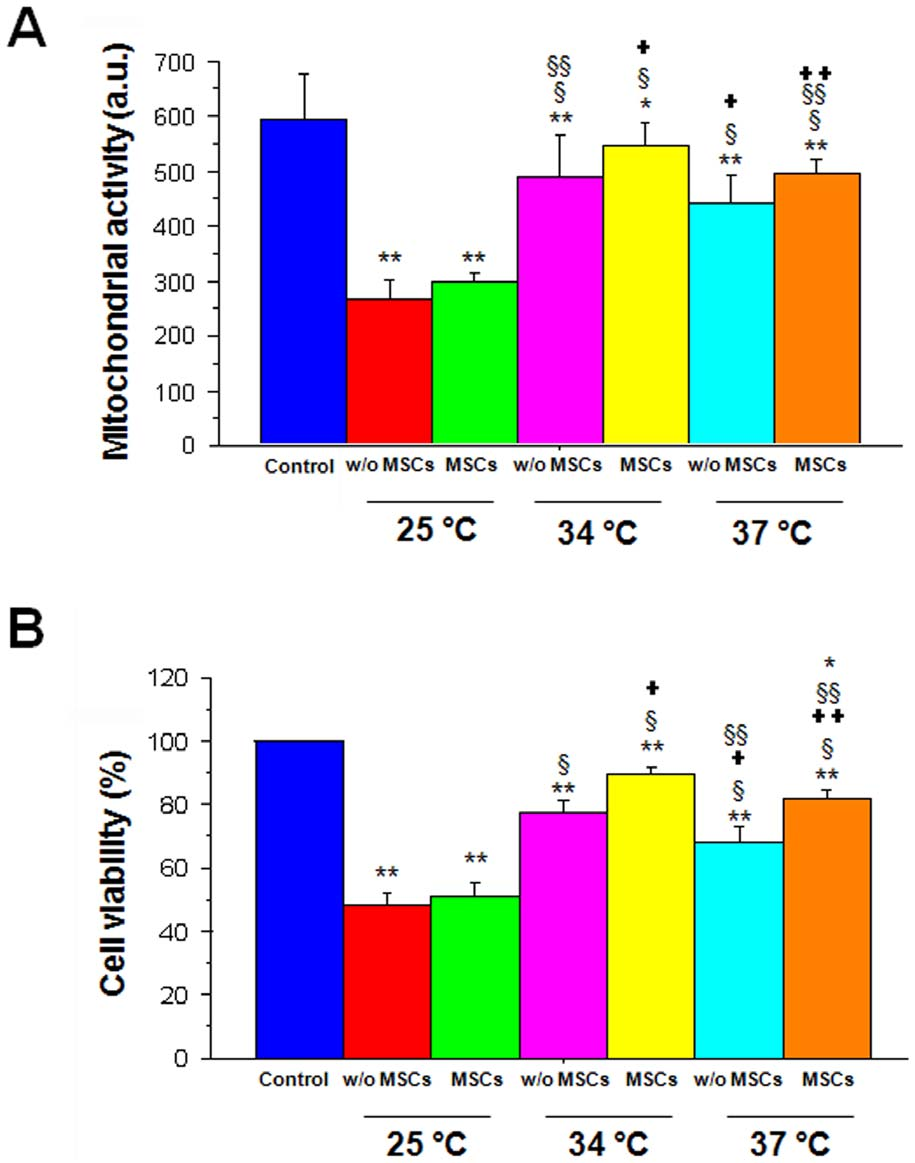
Measurement of cell viability and mitochondrial activity in PRNCs. Moderate hypothermia treatment indicated significant increase cell viability compared to normothermic and sever hypothermia treatments, and enhanced stem cells’ neuroprotection. Black- and white-columns indicate non-implementation and MSCs implementation after 2 hours reperfusion, respectively. Control shows a normoxic condition, atmosphere at 37°C, 95% O_2_, and 5% CO_2_. + Under same temperature condition, *P* value of comparison non-MSCs with MSCs implementation is less than 0.05. *P<0.05: **P<0.01: ***P<0.005. Similarly, moderate hypothermia treatment indicated significant increase in mitochondrial activity compared to normothermic and severe hypothermia treatments, and enhanced stem cells’ neuroprotection. Black- and white-columns indicate non-implementation and MSCs implementation after 2 hours reperfusion, respectively. Cont shows a normoxic condition, atmosphere at 37°C, 95% O_2_, and 5% CO_2_. + Under same temperature condition, *P* value of comparison non-MSCs with MSCs implementation is less than 0.05. *P<0.05: **P<0.01: ***P<0.005. a.u. = absorbance unit.

ANOVA revealed significant treatment effects against OGD (F_6,44_ = 173.007; p<0.0001) (Figure 2). As expected, OGD reduced cell viability as evidenced by the significantly reduced cell viability when the OGD-exposed cells were subsequently introduced to the normothermic condition (p<0.0001 vs. non-OGD controls). Moderate hypothermia blocked OGD-induced cell death and resulted in a significantly higher cell viability compared to normothermic conditions (p<0.0001). Similarly, MSCs alone (under normothermia) suppressed OGD-induced cell death and showed significantly increased cell viability compared to the normothermic condition (p<0.0001). Of interest, the combination treatment of moderate hypothermia and MSCs significantly increased cell viability compared to stem cells alone, moderate hypothermia alone, and normothermia (p’s<0.0001). Conversely, severe hypothermia significantly decreased cell viability following OGD compared to normothermia (p<0.0001). Cell viability remained significantly decreased following severe hypothermia, even in combination with stem cell treatment, compared to normothermia (p<0.0001). Although moderate hypothermia alone, stem cell therapy alone, and a combination of moderate hypothermia and stem cells increased cell viability following OGD, cell viability remained significantly lower than that of the controls (p’s<0.0001).

### Combination Therapy of Moderate Hypothermia and MSCs Normalizes Mitochondrial Activity Following OGD

**Figure 3:**
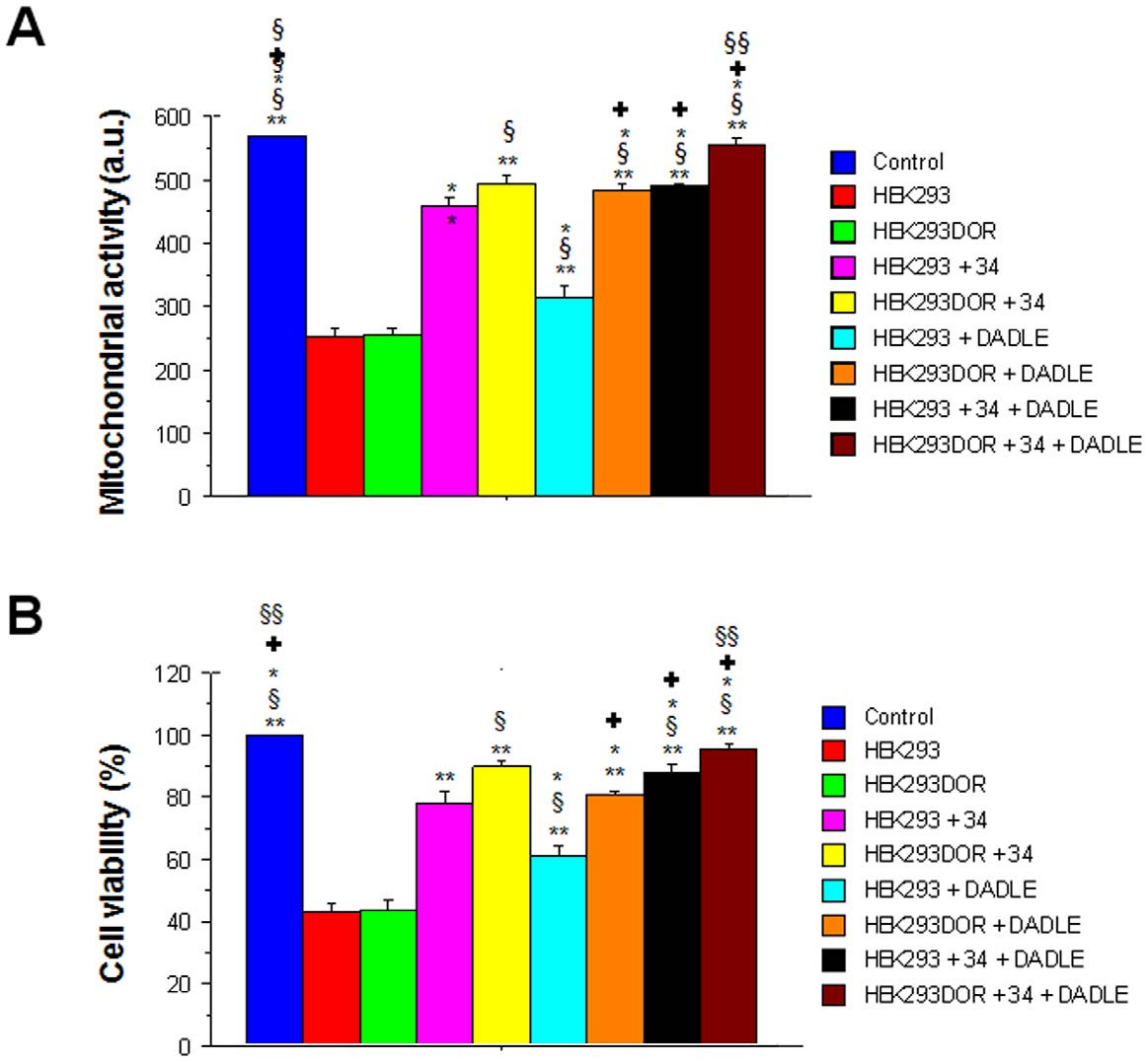
Measurement of mitochondrial activity and cell viability in human embryonic kidney cells (HEK293) and HEK293 cells overexpressing the delta opioid receptor (HEK293DOR). Results revealed that OGD reduced mitochondrial activity and cell viability in HEK293 and HEK293DOR after OGD. Exposure to moderate hypothermia alone, DADLE or combination thereof led to neuroprotection, which was best manifested in HEK293DOR cells. MTT: **p<0.0001 vs. HEK293 and HEK293DOR, §p<0.005 vs. HEK293+34°C; *p<0.005 vs. HEKDOR+34°C; <$>\vskip -1pt\raster="rg1"<$>p<0.0001 vs. HEK293+DADLE; § §p<0.0001 vs. HEK293DOR+DADLE and HEK293+34°C +DADLE. a.u. = absorbance unit.

ANOVA also revealed significant treatment effects on the mitochondrial activity following OGD (F_6,129_ = 100.912; p<0.0001) (Figure 3). As expected, OGD reduced mitochondrial activity when the OGD-exposed cells were subsequently introduced to the normothermic condition (p<0.0001 vs. non-OGD controls). Mitochondrial activity was significantly improved with moderate hypothermia treatment following OGD compared to normothermic condition (p<0.0001). Similarly, MSCs alone (under normothermia) significantly increased mitochondrial activity following OGD (p<0.005). In agreement with the result of the cell viability assay, the combination treatment of moderate hypothermia and MSCs significantly increased mitochondrial activity compared to stem cell therapy alone (p<0.05), moderate hypothermia alone (p<0.005) and normothermia (p<0.0001). Conversely, severe hypothermia significantly decreased cell viability following OGD compared to normothermia (p<0.0001). Mitochondrial activity remained significantly impaired following severe hypothermia, even in combination with stem cell treatment, compared to normothermia (p<0.0001). Although moderate hypothermia alone, stem cell therapy alone, and a combination of moderate hypothermia and stem cells increased mitochondrial activity following OGD, mitochondrial activity remained significantly impaired compared to that of the controls (p<0.05).

### Role of Delta Opioid in Hypothermia-Induced Neuroprotection in Hypoxic-Ischemic-Like Injury *In Vitro*

**Figure 4:**
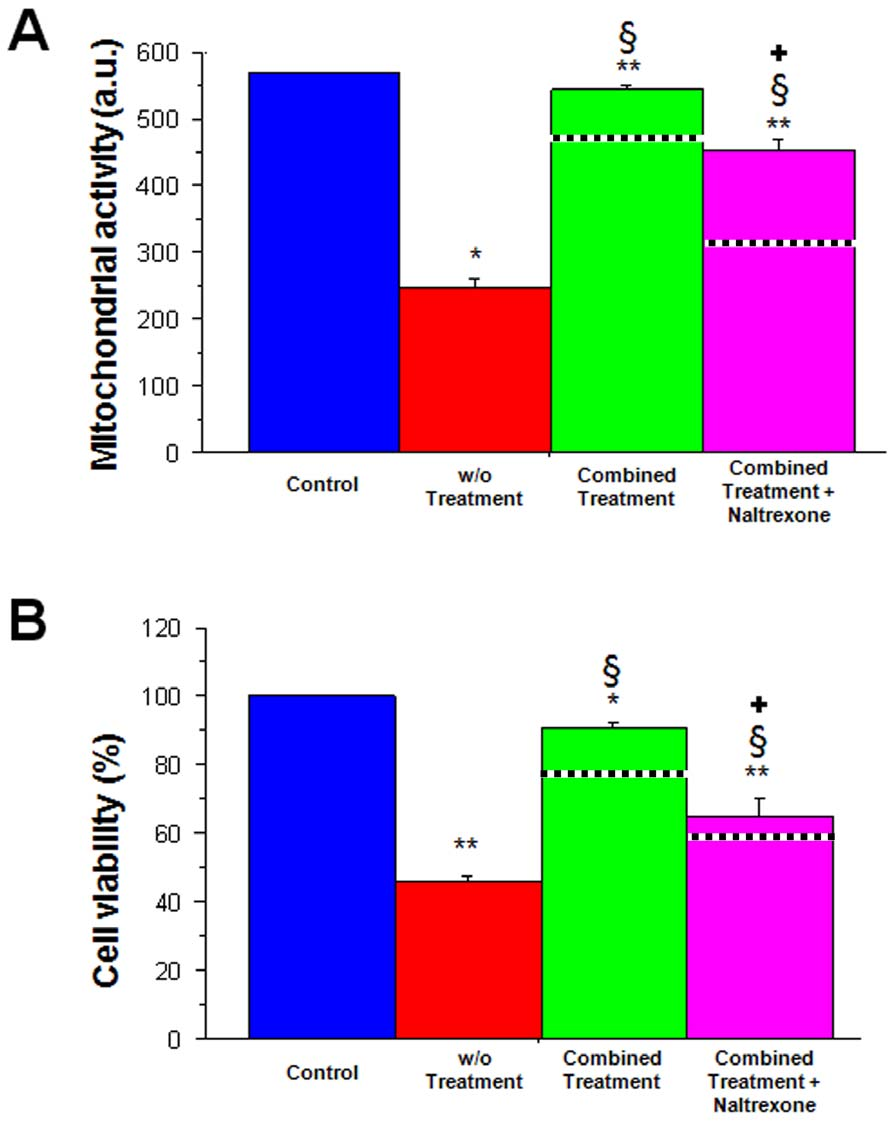
Opioid-mediated neuroprotection of PRNCs exerted by combination therapy of moderate hypothermia, MSCs and DADLE. Results revealed that OGD reduced mitochondrial activity and cell viability in PRNCs under normothermic condition (w/o treatment). The combined treatment, with or without DADLE, significantly improved both mitochondrial activity and cell viability in PRNCs, which was significantly reduced by opioid antagonist naltrexone. MTT: **p<0.0001 vs. control; *p<0.05; §p<0.0001 vs. without treatment; <$>\vskip -1pt\raster="rg1"<$>p<0.0001 vs. combined treatment. Cell Viability: **p<0.0001 vs. control; *p<0.005 vs. control; §p<0.0001 vs. without treatment; <$>\vskip -1pt\raster="rg1"<$>p<0.0001 vs. combined treatment. Dashed lines indicate combined treatment of moderate hypothermia and MSCs without DADLE. a.u. = absorbance unit.

In order to test whether the delta opioid system was involved in hypothermia neuroprotection, we conducted pharmacological manipulations using the delta opioid peptide DADLE and the delta opioid receptor DOR overexpression in HEK 293 cells. ANOVA revealed significant treatment effects against OGD (F_8,18_ = 216.31; p<0.0001) (Figure 4). OGD followed by normothermic condition significantly reduced cell viability (p’s<0.0001 vs. non-OGD controls). Treatment with DADLE significantly increased cell viability similar to the neuroprotection produced by moderate hypothermia, which became more apparent in HEK293DOR cells implicating the delta opioid ligand-receptor signaling pathway as potential mechanism of action (p’s<0.0001). Equally an important finding here is that DADLE in combination with moderate hypothermia resulted in additive effects characterized by near normal (but still lower compared to non-OGD controls) cell viability in both HEK293 and HEK293DOR cells (p’s<0.0001 versus all other treatment conditions).

In a similar manner, ANOVA revealed significant treatment effects on the mitochondrial activity of HEK293 and HEK293DOR cells following OGD (F_8,18_ = 404.88; p<0.0001) (Figure 4). Both cells when exposed to OGD and subsequently to normothermic condition displayed significantly lower mitochondrial activity (p’s<0.0001 vs. non-OGD controls). Interestingly, exposure to DADLE also resulted in therapeutic effects, which again were more pronounced in HEK293DOR cells further providing evidence of the role of the delta opioid ligand-receptor signaling pathway in improving mitochondrial activity after OGD (p’s<0.0001). In agreement with the cell viability results, DADLE in combination with moderate hypothermia showed additive effects as evidenced by significantly elevated mitochondrial activity in both HEK293 and HEK293DOR cells, with the latter achieving non-OGD control level (p’s<0.0001 versus all other treatment conditions).

### Participation of Delta Opioids in Hypothermia and Stem Cell Therapy

Following the demonstration that the delta opioid system was targeted by moderate hypothermia in HEK293 cells, we next embarked on testing DADLE potentiation of combined moderate hypothermia and MSC treatment using the PRNCs. ANOVA revealed significant treatment effects against OGD (F_4,10_ = 248.263; p<0.0001) (Figure 4). Under normothermic condition after OGD, we again found significantly reduced cell viability (p’s<0.0001 vs. non-OGD controls). In contrast, the combination treatment with moderate hypothermia, stem cell treatment and DADLE significantly increased cell viability (p’s<0.0001 vs. OGD controls). To further reveal the role of the delta opioid system, we tried to block the combination therapy effects using naltrexone. Results revealed that naltrexone significantly blocked the cell viability produced by the combination therapy of hypothermia and MSCs with or without DADLE (p’s<0.0001 vs. all treatment condition). However, naltrexone did not completely abolish the therapeutic effects of the combination treatment suggesting a non-opioid mechanism of action (see below). Similar results were obtained from the mitochondrial activity assay. ANOVA revealed significant treatment effects on the mitochondrial activity of PRNCs following OGD (F_4,10_ = 705.528; p<0.0001) (Figure 4). Under normothermic condition after OGD and, we found significantly reduced mitochondrial activity (p’s<0.0001 vs. non-OGD controls). On the other hand, the combination treatment of hypothermia and MSCs with or without DADLE significantly increased mitochondrial activity (p’s<0.0001 vs. OGD controls), which was partially blocked by naltrexone (p’s<0.0001 vs. all treatment condition).

### Non-Opioid Neuroprotective Mechanisms Associated with Hypothermia and Stem Cell Therapy

**Figure 5:**
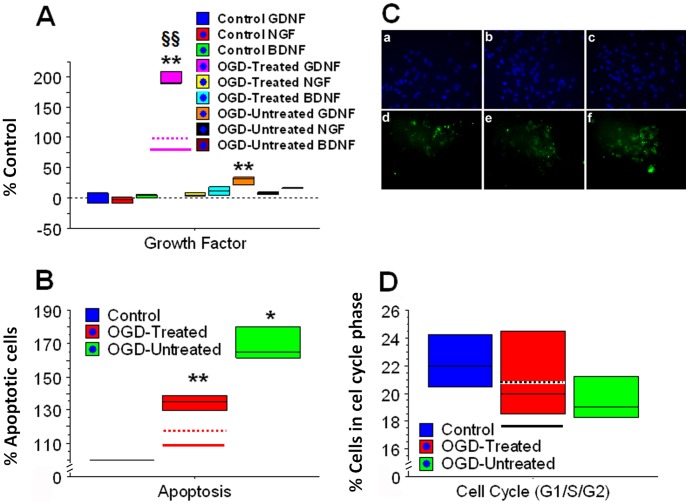
Non-opioid neuroprotective mechanisms accompanying combination therapy of moderate hypothermia, MSCs and DADLE. Results revealed significant upregulation of GDNF, but not NGF or BDNF after combination therapy with or without DADLE (OGD-Treated) compared to OGD-Untreated and Control (Panel A). GDNF was also upregulated slightly in OGD-Untreated condition, likely due to an endogenous compensatory mechanism of the PRNCs. Similarly, OGD-Treated significantly reduced apoptosis compared to Control and OGD-Untreated. Apoptosis via ApoAlert assay is quantified (Panel B) and representative images shown (Panel C) (a-c are Hoechst-stained PRNCs, whereas d-f are corresponding apoptotic stained cells; a/d: Control; b/e: OGD-Treated; c/f: OGD-Untreated). There were no significant differences in cell cycle condition across the three conditions (Panel D). Symbols represent **p<0.0005 vs. Control GDNF; *p<0.0005 vs. Control GDNF; §§p<0.0001 vs. OGD-Treated GDNF (Panel A); **p<0.0001 vs. control; *p<0.005 vs. control and untreated (Panel B). Dashed lines in Panels A, B and D (pink, red, and black, respectively) indicate combined treatment of moderate hypothermia and MSCs without DADLE. Solid lines in Panels A, B and D (pink, red, and black, respectively) indicate treatment with antibody against GDNF and combined treatment of moderate hypothermia and MSCs without DADLE.

That naltrexone did not completely eliminate the therapeutic effects of the combination therapy suggested non-opioid neuroprotective mechanisms. Thus, we next examined non-opioid mechanisms including neurotrophic factor levels in the cell culture media for PRNCs, as well as the cells’ apoptosis and cell cycle status. Among the three growth factors analyzed by ELISA, GDNF exhibited significant elevation in combination therapy of hypothermia and MSCs with or without DADLE compared to other treatment conditions (p<0.0001 vs. controls and OGD-untreated) (Figure 5). Furthermore, treatment with the antibody against GDNF partially blocked the neuroprotection produced by the combination therapy of hypothermia and MSCs with or without DADLE (p<0.05 vs. OGD+combination therapy). Apoptosis was also significantly reduced in the combination therapy of hypothermia and MSCs with or without DADLE (p<0.0001 vs. controls and OGD-untreated). However, we did not obtain significantly different cell cycles in all G1, S1 and G2 phases of PRNCs exposed to the combination therapy of hypothermia and MSCs with or without DADLE (p’s>0.05 vs. controls and OGD-untreated).

## Discussion

The present study demonstrates that moderate hypothermia exerted neuroprotection against experimental hypoxic-ischemic-like injury *in vitro*. Stem cell therapy using MSCs significantly improved the therapeutic outcome of moderate hypothermia. The delta opioid system is shown here to be a key therapeutic pathway solicited by the combination treatment of hypothermia and stem cell therapy. In addition, it appears that hypothermia+MSCs, without DADLE, also utilized the delta opioid pathway because naltrexone reduced the neuroprotection produced by this combination treatment. A non-opioid mechanism, specifically the role of GDNF, implicates a partial involvement of this growth factor the observed benefits. We showed here that the combination treatment of hypothermia+MSCs showed much improved neuroprotective effects compared to stand-alone treatments. A major limitation of the study is that our primary outcome measures of calcein and MTT approximated cell viability and mitochondrial activity at a single time point that warrants additional time-dependent studies.

Despite major scientific breakthroughs in our knowledge of fetal and neonatal pathologies [7], [39]–[41], hypoxic-ischemic injury remains a major cause of brain damage with subsequent neurodevelopmental disabilities, thereby considered a major public health concern [42]. Seizure onset beyond the first 12 hours of life is not only common in newborns with hypoxic-ischemic injury, but also is associated with severe brain injury, suggesting a critical relationship between the timing of onset of neonatal seizure and therapeutic window. Accordingly, any treatment regimen, including hypothermia, is likely to exert benefit if initiated within 5.5 hours after neonatal hypoxic-ischemic injury and continuing over the next 12 or even beyond (i.e., for 72 hours) [43]. The mechanism underlying hypothermia remains elusive, but may include its capacity to reduce oxidative stress, energy deficit, and inflammation [44]. Despite this poorly defined mechanism of action, moderate hypothermia has reached clinical trials and appears to be efficacious in infants with hypoxic-ischemic injury. In the absence of any specific intervention to improve the dismal prognosis of infants with hypoxic-ischemic injury, clinical enthusiasm for a novel treatment is understandable [45].

Our initial goal in this study was to show the therapeutic regimen of hypothermia. The present results indicate that moderate, but not severe hypothermia, was effective in combating hypoxic-ischemic-like injury *in vitro* when compared with normothermia. This neuroprotection was achieved when treatment was initiated at 1.5 hour after OGD and maintained during the reperfusion process, suggesting the direct relevance of hypothermia for clinical application. We recognize that prolonged hypothermia may not be feasible, and may even be detrimental in the clinic. Indeed, patients undergoing aortic arch surgery and kept in prolonged moderate hypothermic circulatory arrest displayed serious side events (i.e., mortality, neurological injury) [46]. Similar complications associated with prolonged hypothermia are seen in the laboratory, in that the rats exposed to experimental cardiac arrest and 11 hours of mild hypothermia display reduced enzyme metabolic capacity leading to elevated drug concentrations and increased adverse drug reaction risk [47]. Accordingly, such adverse effects of prolonged hypothermia prompted us to find an adjunctive treatment in order to produce a stable therapeutic benefit.

Our long-standing interest in stem cell therapy directed our attention to combining moderate hypothermia with MSCs treatment. Various stem cells, derived from embryonic, fetal, adult tissues have been demonstrated to exert therapeutic benefits in hypoxic-ischemic brain injury [12], [48]–[50]. However, no study has yet examined outcomes with combination treatment of moderate hypothermia and MSCs in newborns with hypoxic-ischemic injury. In this study, we showed that moderate hypothermia together with MSC treatment produced enhanced therapeutic benefit when compared with stand-alone treatment of moderate hypothermia or MSCs. This improved outcome produced by the combination therapy may be due to neuroprotective effects afforded by the MSCs in addition to those beneficial actions exerted by moderate hypothermia, namely the ability of MSCs to secrete neurotrophic factors either directly protecting against cell death or inducing the host brain to activate endogenous reparative processes [12], [51], [52]. Neurotrophic factors, such as GDNF, NGF and BDNF, have been shown to play an important role in neurogenesis and angiogenesis [53]. In the same vein, hypothermia may also upregulate growth factors in animals and patients [54]–[56], suggesting that a much more elevated level of these therapeutic substances is afforded by the combination treatment of hypothermia and stem cells.

From a therapeutic standpoint, the combination of hypothermia and stem cell therapy appears as an attractive treatment regimen that can protect the brain of premature infants who are exposed to hypoxic-ischemic-like injury *in vitro*. A conundrum, however, for combining hypothermia with stem cell therapy is identifying a feasible donor cell source that will likely benefit neonatal diseases. A major clinical issue related to therapeutic hypothermia includes the influence of obstetric factors. For example, maternal history, may affect not only encephalopathy but also the infant’s response to interventions [9]. Because of the short therapeutic window in neonates, we postulated here that that the maternal bone marrow-derived stem cells may augment the infant’s response to stem cell therapy in combination of hypothermia allowing a much improved clinical outcome. That the present MSCs augment the hypothermia functional effects lends support to the therapeutic potential of bone marrow-derived stem cells for treatment of hypoxic-ischemic-like injury *in vitro*. Stem cells can be harnessed to thrive in their proliferative and differentiation potential, as well as secretory function, under hypothermic conditions [15], [53]. In the end, hypothermia-induced neuroprotection may benefit from stem cell therapy, and vice versa stem cell fate may be influenced by hypothermia altogether affording a better prognosis in hypoxic-ischemic-like injuries in neonates.

In view of improving the functional outcome of combining hypothermia and stem cells, we posit that finding a mechanistic pathway converging these two treatments may allow a better understanding of neuroprotection. The present results indicate a delta opioid ligand-receptor signaling pathway linking hypothermia and stem cell treatments. Indeed, we showed here that the additive therapeutic effects of the delta opioid peptide DADLE to hypothermia and stem cell therapy were greatly suppressed by the opioid antagonist naltrexone, suggesting the major role of the delta opioid system in neuroprotection. That the DOR-over-expressing kidney cell line enhanced neuroprotection compared to cells without DOR overexpression, and that such therapeutic effect was blocked by naltrexone suggest an opioid receptor mediation of hypothermia and MSCs. Furthermore, naltrexone also prevented neuroprotection produced by hypothermia and MSCs even without DADLE treatment, lending additional evidence of the critical involvement of the opioid pathway in this combination therapy.

The participation of non-opioid neuroprotective processes, including upregulation of neurotrophic factors and stimulation of anti-apoptotic function, in the combination therapy against hypoxic-ischemic-like injury *in vitro* was also demonstrated here. As noted above, MSCs have the capacity to secrete neurotrophic factors and hypothermia can upregulate these growth factors [12], [48], [49], [51]–[53]. In the same fashion, MSCs and hypothermia have been shown to produce anti-apoptotic effects [44], [48], [49]. Both these therapeutic pathways may be inert properties of MSCs and hypothermia, but may also be facilitated by delta opioids outside the ligand-receptor regulatory mechanism, as seen in GDNF upregulation and suppression of apoptotic cell death in DADLE-treated experimental stroke animals [16]. Whereas DADLE has been shown to cause cell cycle arrest in the neural progenitor cell line AF5 [23], the limited cell proliferative features of the present PRNCs (i.e., containing only 15% of astrocytes) might have prevented such cell fate effects of the delta opioid peptide to be manifested.

In summary, the present investigations support the use of combination of moderate hypothermia treatment and of bone marrow-derived stem cell transplantation against hypoxic-ischemic-like injury *in vitro*. Additional experiments are warranted to examine the safety and efficacy of this combination therapy in an animal model of neonatal encephalopathy.
